# Non-invasive cardiac assessment in high risk patients (The GROUND study): rationale, objectives and design of a multi-center randomized controlled clinical trial

**DOI:** 10.1186/1745-6215-9-49

**Published:** 2008-08-01

**Authors:** Alexander M de Vos, Annemarieke Rutten, Hester J van de Zaag-Loonen, Michiel L Bots, Riksta Dikkers, Robert A Buiskool, Willem P Mali, Daniel D Lubbers, Arend Mosterd, Mathias Prokop, Benno J Rensing, Maarten J Cramer, H Wouter van Es, Frans L Moll, Eric D van de Pavoordt, Pieter A Doevendans, Birgitta K Velthuis, Albert J Mackaay, Felix Zijlstra, Matthijs Oudkerk

**Affiliations:** 1 Department of cardiology, University Medical Center Utrecht, Utrecht, The Netherlands; 2 Department of radiology, University Medical Center Utrecht, Utrecht, The Netherlands; 3 Department of radiology, University medical Center Groningen, Groningen, The Netherlands; 4 Julius Center, University Medical Center Utrecht, Utrecht, The Netherlands; 5 Department of radiology, Meander Medical Center Amerfoort, Amersfoort, The Netherlands; 6 Department of cardiology, Meander Medical Center Amersfoort, Amersfoort, The Netherlands; 7 Department of cardiology, St. Antonius Hospital Nieuwegein, Nieuwegein, The Netherlands; 8 Department of radiology, St. Antonius Hospital Nieuwegein, Nieuwegein, The Netherlands; 9 Department of vascular surgery, University Medical Center Utrecht, Utrecht, The Netherlands; 10 Department of vascular surgery, St. Antonius Hospital Nieuwegein, Nieuwegein, The Netherlands; 11 Department of vascular surgery, Meander Medical Center Amersfoort, Amersfoort, The Netherlands; 12 Department of cardiology, University medical Center Groningen, Groningen, The Netherlands

## Abstract

**Background:**

Peripheral arterial disease (PAD) is a common disease associated with a considerably increased risk of future cardiovascular events and most of these patients will die from coronary artery disease (CAD). Screening for silent CAD has become an option with recent non-invasive developments in CT (computed tomography)-angiography and MR (magnetic resonance) stress testing. Screening in combination with more aggressive treatment may improve prognosis. Therefore we propose to study whether a cardiac imaging algorithm, using non-invasive imaging techniques followed by treatment will reduce the risk of cardiovascular disease in PAD patients free from cardiac symptoms.

**Design:**

The GROUND study is designed as a prospective, multi-center, randomized clinical trial. Patients with peripheral arterial disease, but without symptomatic cardiac disease will be asked to participate. All patients receive a proper risk factor management before randomization. Half of the recruited patients will enter the 'control group' and only undergo CT calcium scoring. The other half of the recruited patients (index group) will undergo the non invasive cardiac imaging algorithm followed by evidence-based treatment. First, patients are submitted to CT calcium scoring and CT angiography. Patients with a left main (or equivalent) coronary artery stenosis of > 50% on CT will be referred to a cardiologist without further imaging. All other patients in this group will undergo dobutamine stress magnetic resonance (DSMR) testing. Patients with a DSMR positive for ischemia will also be referred to a cardiologist. These patients are candidates for conventional coronary angiography and cardiac interventions (coronary artery bypass grafting (CABG) or percutaneous cardiac interventions (PCI)), if indicated. All participants of the trial will enter a 5 year follow up period for the occurrence of cardiovascular events. Sequential interim analysis will take place. Based on sample size calculations about 1200 patients are needed to detect a 24% reduction in primary outcome.

**Implications:**

The GROUND study will provide insight into the question whether non-invasive cardiac imaging reduces the risk of cardiovascular events in patients with peripheral arterial disease, but without symptoms of coronary artery disease.

**Trial registration:**

Clinicaltrials.gov NCT00189111

## Background

### Peripheral arterial disease and coronary artery disease

Peripheral arterial disease (PAD) is the term used to refer to lower-extremity arterial disease. It is a sign of systemic atherosclerosis affecting millions of people, in particular the elderly. Reports from the Framingham Heart Study suggest that the prevalence of PAD has increased between 1970 and 2003 [1,2]. Estimates are that approximately 10% of individuals > 55 years have asymptomatic PAD (defined as an ankle-brachial index (ABI) < 0.90) [3]. The prevalence of so called intermittent claudication (IC) in patients aged 55 to 74 years is approximately 4.6% [3] and the prevalence of pain at rest and necrotic lesions (Fontaine stage IV) is approximately 1% [4]. Despite the relatively benign prognosis for the affected limb, symptoms of IC should be regarded as a sign of systemic atherosclerosis and a high risk of cardiovascular events. In a review on IC, Coffman et al. [5] described survival rates among IC patients of approximately 70% to 80% after 5 years, 40% after 10 years, and 26% after 15 years. More recent studies showed an overall 5-year-mortality rate of 19.2% vs. 10% in controls [6] and 10-year-mortality rates of 61.7% among male and 33.3% among female patients with IC, compared to 16.9% of men and 11.6% of women without evidence of PAD [7]. The mean age of participants in these studies was 67 and 66 years, respectively. Mortality due to coronary artery disease (CAD) after 5 years in a study by Leng et al. was 5.5% vs. 2.6% in controls [6] and after 10 years cardiac death occurred in 35.3% of men and 9.1% of women with IC, compared to 5.5% of men and 2.2 % of women without IC [7]. So not only do PAD patients have two or three times the overall mortality, the risk of cardiac death is even 4–6 times higher [7].

This increase in cardiovascular mortality is not surprising since several studies showed a two or three times increase in cardiovascular morbidity in PAD patients [6,8,9]. In 2003 Sonecha et al. published a study in which they found CAD in 46% of IC patients, compared to 6% in controls; 31% of claudicants even had 2-/3-vessel disease [10]. Aronow et al. found a prevalence of CAD in PAD patients of 58% [11]. Hertzer et al. described the results of coronary angiography (CAG) in 1000 patients scheduled for vascular surgery. In this group, 381 had complaints of lower extremity ischemia, of whom 166 (44%) had no cardiac complaints. CAG revealed the presence of CAD in 86% of these cardiac asymptomatic PAD patients [12]. Therefore, assessment of cardiac atherosclerotic abnormalities using non-invasive techniques followed by appropriate treatment may help to improve survival in patients with PAD but yet without cardiac symptoms.

### Cardiac imaging with multi-detector CT and MRI

Since the discovery of selective coronary angiography (CAG) by Sones in 1958, it has been the method of choice for detection and follow-up of CAD. Several studies have shown that diagnostic CAG has a morbidity of 2% and a mortality of approximately 0.1% [13-15]. For screening purposes non-invasive imaging would be much more suitable. The rapid development of multi-detector computed tomography (MDCT) has made it possible to image the heart and its coronary arteries in a non-invasive way. It is much faster than older scanners and images are obtained with sub millimeter spatial resolution and high temporal resolution. As a result of simultaneous recording of an electrocardiogram (ECG) signal, several image reconstructions are possible in different phases of the heart cycle [16].

Several studies showed the high accuracy of MDCT to detect clinically important coronary stenoses. [17-19] Not only the costs and risk of complications are lower with MDCT than with CAG [13-15], this technique also has the advantage of vessel wall visualization. Both the composition of the plaque and its impact on the vessel lumen can be detected. A distinction can be made between lipid, fibrous and calcified coronary plaques [20]. In recent years it has become clear that plaque composition may be a better risk-predictor for acute coronary events than stenosis grade. Rupture of so called vulnerable plaques accounts for approximately 70% of sudden coronary deaths[21]. Although the absolute risk of severely stenotic plaques may be higher than the absolute risk of mildly stenotic plaques, the number of plaques with mild stenoses overwhelmingly exceed the number of plaques with severe stenoses [21].

Dobutamine stress cardiovascular magnetic resonance imaging (DSMR) is used to identify wall motion abnormalities of the left ventricle indicative of myocardial ischemia [22-26]. It has been shown to be an accurate and safe diagnostic modality to assess myocardial ischemia and viability in patients with proven or suspected CAD [23-28]. A study by Nagel et al. showed that the presence of myocardial ischemia can be detected more accurately with DSMR than with dobutamine stress echocardiography (DSE). Image quality of DSMR is higher and with MRI sensitivity increased from 74.3% to 86.2% (P < 0.05) and specificity increased from 69.8% to 85.7% (P < 0.05) compared to echocardiography [23]. With the use of myocardial tagging sensitivity can be increased up to 96% [26]. In this study by Kuijpers et al. the cardiovascular occurence-free survival rate was 98.2% after a negative DSMR during a mean follow-up of 17.3 months. Furthermore, MRI allows optimal detection of dysfunctional, but viable myocardium. This is of clinical importance since revascularization of dysfunctional, but viable myocardium may improve left ventricular function and thus prognosis [29].

In patients with non-specific symptoms of coronary artery disease DSMR can be used to assess risk levels for coronary events with high accuracy. In a group of 100 patients suspected of coronary ischemia Van Dijkman et al. found a positive predictive value (PPV) of 98% and also a negative predictive value (NPV) of 98% for ischemia with DSMR. In this study the prevalence of ischemia was 43% [30]. In another study by Hundley et al. a 97% cardiac event free survival rate in 103 patients suspected of ischemia with a negative DSMR was observed [25]. Patients with a negative DSMR without rest wall motion abnormalities (RWMA) and without a history of CAD have an excellent cardiac prognosis and can be excluded from further clinical follow-up [31].

Compared to other non-invasive techniques, DSMR may be a valuable adjunct for the assessment of patients with (suspected) ischemic heart disease [32].

### Treatment of silent coronary artery disease

According to the guidelines CABG or PCI may be considered as first line therapy in case of severe abnormalities in the coronary artery tree, even in asymptomatic patients [33]. Absence of cardiac symptoms should not be regarded as a sign of a more benign process [34]. In addition, silent myocardial ischemia has been shown to increase coronary artery disease risk and evidence indicates that in certain groups of these patients CABG or PCI treatment may reduce the risk. The results of the Asymptomatic Cardiac Ischemia Pilot (ACIP) study indicate that higher-risk patients with asymptomatic ischemia and clinically important coronary artery abnormalities, who undergo revascularization with CABG or PCI may have a better outcome as compared to those only receiving medical therapy [35]. Studies on the treatment of silent ischemia are all conducted in small groups of patients with coronary abnormalities [36]. In patients with left main disease, the survival benefit of CABG compared to medical therapy is 19.3 months at 10-year follow-up. Therefore, the benefit of surgery over medical treatment for patients with left main stenosis (> 50%) is little argued [37].

## Study objective

**Figure 1:**
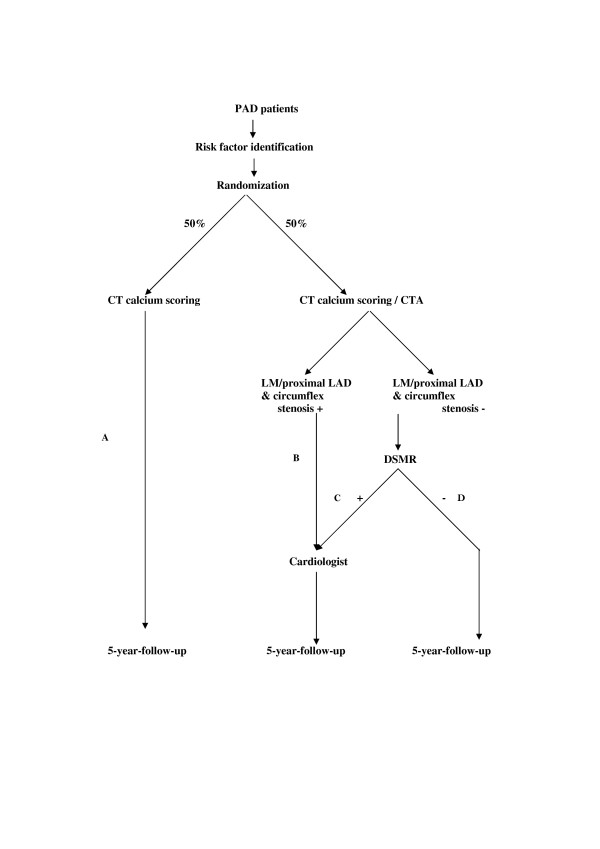
Flow Diagram of the GROUND Study.

This prospective, randomized, controlled, multicenter trial is designed to evaluate whether a cardiac imaging algorithm using non-invasive imaging techniques followed by evidence based treatment will reduce the risk of cardiovascular disease in cardiac asymptomatic patients with peripheral arterial disease. This imaging algorithm consists of coronary calcium scoring, MDCT angiography, and dobutamine stress MRI. Participants will be followed up for a period of five years (figure 1). As a secondary objective we will explore the role of coronary calcification in this particular patient group.

## Methods

### Study group

The study group will consist of approximately 1200 patients with peripheral arterial disease without a history of symptomatic cardiac disease. Patients are recruited from the vascular surgery departments of the participating centers (appendix 1). The study is in compliance with the Helsinki Declaration and local ethics committees gave their approval. Patients willing to participate will be asked to sign the informed consent form. Patients are eligible if they are 50 years or older and have peripheral arterial disease, at least stage Fontaine II, as diagnosed by the vascular surgeon. Patients will be considered not eligible for the study if they meet one of the following exclusion criteria: physician diagnosed history of symptomatic cardiac disease; cardiac rhythm other than sinus; unable to sustain a breath-hold for 25 seconds; asthma; contra-indications to MRI examination, such as vessel clips in the brain, metal splinters in the eye, insulin pump or other electronical devices that cannot be removed easily, metal implants, port-a-cath catheter or claustrophobia; contra-indications to iodinated contrast agent; severe arterial hypertension (> 220/120 mmHg); clinically important aortic stenosis; unable to remain in supine position for at least 60 minutes; extreme obesity (BMI > 40 kg/m^2^); renal insufficiency (serum creatinine level exceeding 140 μmol/l); severe physical deterioration due to concomitant disease; language barrier; and contra-indications to dobutamine.

### Baseline risk factors

At baseline, eligible patients complete a questionnaire on current medication use, risk factors and quality of life. Height, weight, blood pressure and ankle pressure for calculation of the ankle-brachial index will be measured in the outpatient clinic. Total cholesterol, high density lipoproteins (HDL), triglycerides, creatinine, homocysteine, glucose and high sensitivity c-reactive protein will be measured at the local laboratory. These measurements were not standardized across the four different laboratories. To prevent further progression of their present cardiovascular disease all patients will be treated according to the Dutch guidelines for treatment of atherosclerotic peripheral arterial disease [38]. These guidelines state that these patients should receive aspirin, a statin and antihypertensive medication, if indicated. Patients also receive proper advice regarding exercise, healthy diet and cessation of smoking.

### MDCT imaging

All centers participating in the GROUND study are experienced in making cardiac MDCT angiography scans and use at least a 16-slice CT scanner. The patient preparation will start with explanation of the procedure. The ECG monitor is connected and sinus rhythm is monitored for 1 minute. Then the patient practices a 25 second breath-hold. The MDCT calcium scoring examination will follow a scout view. It will be done either prospectively ECG-triggered or retrospectively gated according to the local hospital protocol. For the patients randomized to the imaging arm of the study, calcium scoring will be followed by the contrast enhanced retrospectively ECG-gated CT angiography. An 18 G intravenous line is started. Patients who have a heart rate over 60 bpm will be administered i.v. beta-blockers (minimum of 5 mg metoprolol). Patients will continue to receive beta-blockers until their heart rates are below 60 bpm, or 20 mg has been administered. Blood pressure will be monitored. The contrast volume and infusion rate will be calculated individually depending on patient weight and scan duration and contrast concentration. Ten axial image datasets will be reconstructed every 10% of the RR interval on the electrocardiogram.

Total examination time for CT calcium scoring will be approximately 10 minutes and the total examination time for CT calcium scoring and CT angiography, including preparation of the patient, will be approximately 30 minutes. Total estimated radiation dose for the patient is recorded.

### MDCT Analysis

The cardiac MDCT data will be analyzed by the site investigator at the site. The reader will use the appropriate workstation for all data analysis. The site investigator is responsible for incidental abnormal findings in the dataset.

The calcium scoring study will be evaluated using an established analysis program (Heartbeat-CS, EBW, Philips Medical Systems). Agatston, Mass and Volume scores will be determined and recorded on case report forms. For the MDCT angiography the site investigator will identify the phase with the least amount of cardiac motion. This phase is then loaded into the appropriate application. Depending on the coronary morphology and quality of the scan several post processing techniques will be applied to assess the coronary arteries. The dataset will be evaluated in terms of contrast opacification, assessability, stenoses and plaques. The proximal, medial and distal coronary arteries and cranial, medial and caudal right ventricle are evaluated for contrast opacification and image noise using a 5-point scale (1 = non-diagnostic, 2 = limited diagnostic, 3 = acceptable, 4 = good, 5 = excellent).

The 15-segment tree from the American Heart Association will be used for segment definition. Each segment will be evaluated for assessability using a 5-point scale (1 = non-diagnostic, 2 = limited diagnostic, 3 = acceptable, 4 = good, 5 = excellent). For the segments that are not assessable (score 1 or 2) the reader will indicate why the segment cannot be evaluated according to the following choices: (0) anatomical reason, (1) respiratory motion, (2) cardiac motion, (3) arrhythmia, (4) calcium, (5) vessel size (small caliber), (6) poor opacification, (7) streak artifacts, (8) scan range, (9) noise, (10) technical failure. Any luminal narrowing greater than 30% will be visualized from the curved MPR and will be quantified according to a 4-point scale: (1) 30–50%, (2) 50–70%, (3) 70–99% and (4) 100%. The type of visualized plaque will be indicated as soft, calcified or mixed. All scan data will be transferred to the core laboratory for a second reading of the data. Inter- and intra-observer variability will be determined.

### DSMR imaging

Patients randomized to the imaging group will under go dobutamine stress MRI within three weeks of the MDCT angiography. All beta-blocking medication will be stopped 4 days prior to the examination [26]. After instructions by the technician, the patient is positioned on the scanning table and an intravenous access will be established through an antecubital vein. ECG leads, a phased-array surface coil covering the heart, and a brachial blood pressure cuff are applied. During the procedure, a single-lead ECG will be continuously monitored. Systolic blood pressure, diastolic blood pressure and heart rate will be recorded at baseline and every three minutes throughout the procedure.

Baseline imaging will consist of acquiring three short-axis cine images (basal (1.5 cm below mitral valves), mid-ventricular and apical) and one vertical long-axis cine image. Cine tagged images are made in the three short axis planes.

During the DSMR dobutamine will be infused intravenously using a digital pump injector situated outside the scanner room. The dose will be increased to 10, 20, 30 and 40 μg/kg/min with a six minutes time interval. In case of rest wall motion abnormalities (RWMA), infusion will be started at 5 μg/kg/min. Image acquisition will start three minutes after each dose increment. Imaging will consist of acquiring three short-axis cine images (basal, mid-ventricular and apical) and one vertical long axis with and without myocardial tagging. Criteria for ending the examination are (1) development of new or worsening wall motion abnormalities (NWMA) in more than 1 myocardial segment, (2) fall of systolic blood-pressure of > 40 mmHg, (3) marked hypertension > 240/120 mmHg, (4) severe chest pain, (5) complex cardiac arrhythmia's and (6) intolerable side effects of dobutamine.

Both a radiologist (or a trained radiology resident) and a cardiologist (or a trained cardiology resident) will be present in the MR suite to monitor the condition of the patient and to directly evaluate the images. The target heart rate rule is not applied. Studies have shown that this is a safe and effective method [26,28,31].

All participating centers have experience in dobutamine stress testing. Although side effects are rare, a protocol to remove the patient from the scanner room in case of an emergency is practiced regularly. Total examination time for a DSMR study, including preparation of the patient, will be approximately 50 minutes.

### DSMR Analysis

The DSMR data will be analyzed by the site investigator at the site. The reader will use the appropriate workstation for all data analysis. For image interpretation multiple cine loop display will be used displaying at least three different stress levels for each slice simultaneously. Per segment wall motion will be graded using a 4-point scale according to the guidelines of the American Society of Echocardiography (1 = normal or hyperkinesia, 2 = hypokinesia, 3 = akinesia and 4 = dyskinesia). The sum of points is divided by the number of analyzed segments and yields the wall motion score [39]. Normal contraction results in a wall motion score of 1, a higher score is indicative of wall motion abnormalities. During dobutamine stress with increasing doses, a lack of increase in either wall motion or systolic wall thickening, a reduction of both or significant changes in the rotational pattern of left ventricular myocardium ('tethering') are indicative of pathological findings. Myocardial ischemia will be defined as an induced WMA in at least two segments at consecutive planes of the left ventricle. RWMA will be defined as WMA in one or more segments at baseline. If RWMA's are present, which improve during low-dose dobutamine stress, but worsen during peak-stress, this will be considered diagnostic of inducible myocardial ischemia. If RWMA's are present, which do not improve with low-dose dobutamine, this will not be considered diagnostic of inducible ischemia. Also ejection fraction, end-diastolic volume and end-systolic volume will be documented. All scan data will be transferred to the core laboratory for a second reading of the data. Inter- and intra-observer variability will be determined.

### Randomization

Randomization will be performed per hospital to ensure an equal distribution of groups of patients within hospitals. Directly after the patient gives informed consent, he or she is randomized with the use of the randomization module at the GROUND website. Only the data management center is aware of block size. This way half of the patients will be randomized for the imaging-with-treatment algorithm (groups B, C and D, figure 1), the other half of the patients (group A) undergoes only CT calcium scoring and enters follow-up. Patients randomized for the treatment groups (groups B, C and D, figure 1) will be scheduled for MDCT angiography. If a stenosis of the left main coronary artery (LM) (or equivalent) of more than 50% is observed on the CTA of a patient in the imaging-with-treatment group, he/she will be referred to a cardiologist for further diagnosis and treatment (group B, figure 1). A stenosis in the proximal left anterior descending coronary artery (LAD) in combination with a stenosis in the proximal circumflex coronary artery (LCX) is considered equivalent to a LM stenosis. All other patients in this group will undergo DSMR testing (groups C and D). Patients with a DSMR positive for ischemia are referred to a cardiologist for further diagnosis and treatment (group C). Further diagnostics and treatment will be left up to the cardiologist.

### Data collection

Study data, including detailed data on diagnostic and therapeutic measures taken by the cardiologist, will be collected on case report forms (CRF's) and submitted on line to the data management center, located at the Julius Center for Health Sciences and Primary Care , where all forms are reviewed for completeness. CRF's are available on the GROUND website. Data will end up in a dedicated database.

### End points and follow-up

All patients will be asked to fill out a short follow up form every half year for a total period of five years. The occurrences of events are recorded. The quality of life assessment is based on the SF36 questionnaire and repeated after 12, 30, 48, and 60 months. This questionnaire has been validated in the Dutch population [40]. Endpoints of the GROUND study are in concordance with the SMART [41] study endpoints.

The term 'end points' is used to describe death, cardiovascular complications, and interventions. Apart from death the occurrence of an endpoint does not imply that the follow-up will be ended. Endpoints in the GROUND study are summarized as MACE: Major Adverse Clinical Events. Primary outcome is a composite endpoint comprising fatal and non-fatal myocardial infarction and stroke, and vascular death (death due to vascular disease). Secondary end points are: fatal and non-fatal myocardial infarction; fatal and non-fatal stroke; vascular interventions; amputation; aortic rupture; end stage renal failure extra cranial hemorrhage; complications of CABG or PCI and all cause mortality.

Reported endpoints are classified by the Endpoint Committee, which is unaware of the randomization allocation. Clinical information (letters of discharge) is obtained from the treating specialist or general practitioner. All reported endpoints enter an endpoint verification procedure. Copies of discharge records are sent to the members of the Endpoint Committee. The members of the Endpoint Committee do not share the information between each other, but classify the events independently. Only if discharge records are inconclusive further medical information is obtained (results from laboratory findings, copy of the ECG, copies of imaging reports). The classifications are compared. If two members do not agree the endpoint will be discussed with the blinded research physicians of the GROUND study group. They will decide or consult an extra physician, whose judgment is regarded as final.

### Sample size considerations

The sample size is determined by the estimated risk in the group of patients randomized to usual care (control group, A, figure 1) and the risk observed in the groups that undergo cardiac imaging followed by subsequent treatment by a cardiologist as outlined in the protocol (groups B to D). Based on earlier studies in the Netherlands the 5-year-risk in the control group is assumed 24%[42]. The 5 year risk of cardiovascular morbidity and mortality among the IC patients that undergo cardiac imaging and subsequent treatment is based on the sum of **I) **the risk observed in those with stenosis in the main left coronary artery (group B, figure 1) plus **II) **the risk observed in those with limited vessel disease but no ischemia during the dobutamine stress test (group D) plus **III) **the risk observed in those with limited coronary vessel disease but with ischemia during the dobutamine stress test (group C).

The prevalence of these subgroups B to D in the arm of cardiac imaging and subsequent treatment is estimated to be 8% for B; 70% for D and 22% for C. Using the literature we have estimated the risk of cardiovascular morbidity and mortality for these groups of patients belonging to category B to D.

The 5-year risk of cardiovascular morbidity and mortality in these subgroups is 66% for those with stenosis of the left main coronary artery[41] (group B), 32.8% for those with cardiac ischemia during the stress test (group C) and 16.4% for those without cardiac ischemia (group D).

The estimates for patients in categories C and D are based on the assumption that those with cardiac ischemia have a doubling of risk compared with those without cardiac ischemia and that the risk of all groups combined should add up to 24%.

The effects of interventions performed by the cardiologist on the risk observed in PAD patients who undergo cardiac imaging are based on published international data from the ACC and AHA guidelines. These effects are for the two appropriate subgroups: 70% reduction in 5-year event rate using reperfusion therapy (PCI/CABG) for group B and 40% reduction in 5-year event rate using reperfusion therapy (PCI/CABG) for group C. Patients without cardiac ischemia (group D) will receive no treatment.

Based on these estimates, the 5-year risk of cardiovascular morbidity and mortality will be 17.4 % in the intervention group (the combined risk for groups B and C; 70% event reduction in the group with a risk of 66% applying to 8% of the population; 40% event reduction in the group of patients with a risk of 32.8% applying to 22% of patients), reflecting an estimated relative reduction in cardiovascular morbidity and mortality of 24%.

The total number of patients randomized to achieve this goal, with a two-sided alpha of 0.05 and 80% power will be 1222. For this calculation we used dedicated software ('Power', dr. P.G.H. Mulder, Erasmus Medical Center, Rotterdam, the Netherlands). A study of this size has a statistical power of 80% at a two-sided alpha level of 0.05. The reason for using interim analysis [43] is that on average fewer patients are needed in the study when the expected difference in the primary outcome variable is real or when no difference can be expected anymore, therefore increasing efficiency. Sequential analyses are performed on survival outcome variables according to the double triangular test as described by Whitehead [44] and implemented in the computer program PEST version 4 [45]. The sequential (interim) analyses will be performed every three months by an independent data safety monitoring board (DSMB).

### Statistical analysis

The analyses will be performed using the intention-to-treat principle. To assess whether intervention is related to a reduced risk of events we will use a chi-square analysis comparing the observed absolute risks across treatment groups. In addition, treatment efficacy will be assessed using a Cox regression model and expressed as a hazard ratio with corresponding 95 % confidence intervals. Since this is a randomized controlled trial, no adjustments will be made, although centers may be added. Associations will be considered significant at p < 0.05. All statistical tests will be 2 sided. For statistical analyses we will use SPSS (SPSS for Windows, Chicago, Ill, SPSS Inc.).

## Conclusion

Peripheral arterial disease is a common disease among elderly persons and is associated with a very high risk of cardiovascular events. In this study patients with peripheral arterial disease, but without cardiac symptoms, are randomized to an imaging arm consisting of multi-detector CT angiography of the coronary arteries and dobutamine stress MRI or to a control group in which case only a coronary calcium CT scan will be performed at baseline. In case of a positive finding in the imaging arm, patients are referred to a cardiologist who will take appropriate action. All participating patients will enter a 5-year follow-up for the occurrence of cardiovascular events. To the best of our knowledge GROUND is the first large trial designed to assess the value of multi-detector CT and MRI stress testing in reducing the morbidity and mortality of patients with peripheral arterial disease but yet without cardiac symptoms. At the time of writing this manuscript all centers are actively enrolling patients. The first patient enrolled in January 2005. The number of included patients is currently 228.

## Competing interests

The authors declare that they have no competing interests.

## Authors' contributions

AdV and AR drafted the manuscript, MB contributed to the statistical analysis section. WM, MP, MB, BR, MO and FZ were the initiators of the study, contributed to the design of the study and revised the manuscript critically. AdV, AR, HZ obtained local ethics approval. HZ, RD, RB, DL, AM, MC, HE, AdV and AR recruited patients and were responsible for the scanning. FM, EP, PD, BV, and AJM contributed to the design and coordination of the study, helped to draft the manuscript and revised it critically. All authors read and approved the final manuscript.

## Appendix

### Participating Centers

The following hospitals in the Netherlands have agreed to participate in the GROUND-study: University Medical Center Groningen; University Medical Center Utrecht; St. Antonius Hospital Nieuwegein; Meander Medical Center, Amersfoort.

### Executive Committee

The Executive Committee is responsible for the design of the GROUND study. It will coordinate and direct the study to ensure its overall success and decide on practical issues concerning the study. It will act upon recommendations of the Data Safety and Monitoring Board regarding continuation of the study.

Members of the executive committee are:

Prof. W.P.Th.M. Mali, MD, PhD, University Medical Center Utrecht, responsible for the trial coordination

Prof. M. Oudkerk, MD, PhD, University Medical Centerl Groningen, responsible for the radiological coordination

Prof. F. Zijlstra, MD, PhD, University Hospital Groningen, responsible for the cardiological coordination

Dr. M.L. Bots, MD, PhD, University Medical Center Utrecht (Julius Center) responsible for the epidemiological coordination (project management, general data base management and statistical analyses)

### Steering Committee

The steering committee consists of radiologists, cardiologists, epidemiologists, vascular surgeons and researchers of the participating centers. The Steering Committee will perform the actual imaging procedures of the study. Members will inform the Executive Committee on the progress of the study regularly. The steering committee and the executive committee will meet twice annually.

Members of the steering committee are: Mathias Prokop, MD, PhD; Pieter A. Doevendans, MD, PhD; Annemarieke Rutten, MD; Alexander M. de Vos, MD; Frans Moll, MD, PhD; Evert-Jan Vonken, MD, PhD; Maarten-Jan M. Cramer, MD, PhD; Birgitta K. Velthuis, MD, PhD (University Medical Center Utrecht); Hester J. van der Zaag, MD; R.A. Tio, MD, PhD; T.P. Willems, MD, PhD; P.M. van Ooijen, PhD; R. Vliegenthart, MD, PhD (University Medical Center Groningen); Benno J. Rensing, MD, PhD; H. Wouter van Es, MD, PhD; H.D. van de Pavoordt, MD, PhD (St. Antonius Hospital Nieuwegein); Arend Mosterd, MD, PhD; Ben G. Heggelman, MD; Robert A. Buiskool, MD; Albert J. Mackaay, MD, PhD (Meander Medical Center Amersfoort).

### Endpoint Committee

The Endpoint Committee will systematically evaluate suspected endpoints. Members are: F. Zijlstra, MD, PhD (cardiologist); B. Rensing, MD, PhD (cardiologist); A. Mosterd, MD, PhD (cardiologist); J. de Keyser, MD, PhD; W.J. Schonewille, MD (neurologist); T.W.M. Raaijmakers, MD, PhD (neurologist); M.L. Bots, MD (epidemiologist), PhD; A. Rutten, MD (radiologist in training); A.M. de Vos, MD (cardiologist in training).

### Data Safety and Monitoring Board

The data safety and monitoring board performs statistical analyses of un-blinded interim data and formulates recommendations for the Steering Committee on the continuation of the trial. The DSMB may also offer unsolicited recommendations on the continuation of the trial, for example after publication of results of similar trials. Every three months the chair of the DSMB will be provided an interim dataset to perform sequential analyses. When appropriate, given the results from the interim analysis, the chair will call for a meeting with the other DSMB members. Members of the Data Safety and Monitoring Board are: Ingeborg van der Tweel, PhD (statistician); Maarten-Jan M. Cramer, MD, PhD (cardiologist); Hester J. van der Zaag, MD, PhD (epidemiologist); Diederick E. Grobbee, MD, PhD (epidemiologist).
